# 256-Slice CT Angiographic Evaluation of Coronary Artery Bypass Grafts: Effect of Heart Rate, Heart Rate Variability and Z-Axis Location on Image Quality

**DOI:** 10.1371/journal.pone.0091861

**Published:** 2014-03-17

**Authors:** Bettina M. Gramer, Patricia Diez Martinez, Anne S. Chin, Marie-Pierre Sylvestre, Sandra Larrivée, Louis-Mathieu Stevens, Nicolas Noiseux, Gilles Soulez, Ernst J. Rummeny, Carl Chartrand-Lefebvre

**Affiliations:** 1 Radiology, University of Montreal Medical Center (CHUM), Montreal, Quebec, Canada; 2 Research Center of the University of Montreal Medical Center (CRCHUM), Montreal, Quebec, Canada; 3 Department of Diagnostic and Interventional Radiology, Klinikum rechts der Isar der Technischen Universität München, Munich, Germany; 4 Department of Social and Preventive Medicine, University of Montreal, Montreal, Quebec, Canada; 5 Cardiac Surgery, CHUM, University of Montreal and CRCHUM, Montreal, Quebec, Canada; University of Groningen, Netherlands

## Abstract

**Purpose:**

The objective of this study is to assess the effect of heart rate, heart rate variability and z-axis location on coronary artery bypass graft (CABG) image quality using a 256-slice computed tomography (CT) scanner.

**Methods:**

A total of 78 patients with 254 CABG (762 graft segments) were recruited to undergo CABG assessment with 256-slice CT and prospective ECG-gating. Two observers rated graft segments for image quality on a 5-point scale. Quantitative measurements were also made. Logistic and cumulative link mixed models were used to assess the predictors of graft image quality.

**Results:**

Graft image quality was judged as diagnostic (scores 5 (excellent), 4 (good) and 3 (moderate)) in 96.6% of the 762 segments. Interobserver agreement was excellent (kappa ≥0.90). Graft image quality was not affected by heart rate level. However, high heart rate variability was associated with an important and significant image quality deterioration (odds ratio 4.31; p  =  0.036). Distal graft segments had significantly lower image quality scores than proximal segments (p ≤ 0.02). Significantly higher noise was noted at the origin of the mammary grafts (p  =  0.001), owing to streak artifacts from the shoulders.

**Conclusion:**

CABG imaging with 270-msec rotation 256-slice CT and prospective ECG-gating showed an adequate image quality in 96.6% of graft segments, and an excellent interobserver agreement. Graft image quality was not influenced by heart rate level. Image quality scores were however significantly decreased in patients with high heart rate variability, as well as in distal graft segments, which are closer to the heart.

## Introduction

Each year, approximately 300 000 coronary artery bypass graft (CABG) surgeries are performed in the US [1]. CABG aims to restore adequate blood supply to the ischemic heart, and the success of the operation depends mainly on the patency of the bypass grafts [2]. While catheter angiography remains the gold standard for coronary and CABG lumen evaluation, it is still associated with a 0.11% risk of death, 0.05% risk of myocardial infarction, 0.07% risk of stroke, and 0.43% risk of vascular complications [3], [4]. The risk of complications is increased in patients with CABG [5]. CABG catheterization is technically more difficult than for native vessels, and the location of the bypass ostia is variable; the procedure has a longer duration, and more contrast agent is used [5]. It is obviously desirable to avoid costly and risky invasive cardiac procedures for CABG patients.

Advances in multidetector computed tomography (MDCT) technology have shown an increasingly accurate non-invasive CABG assessment [6]–[8]. Since grafts are less influenced by cardiac motion, have a wider luminal diameter, and are less calcified, MDCT can be more accurate for graft imaging than for native coronary vessels [5]. Using 16- and 64-slice systems, MDCT has excellent pooled sensitivity and specificity values for graft stenosis (≥ 50% in diameter) detection (respectively, 94.4 and 98.0%) [5]. Sensitivity and specificity values are even better for occlusion detection (respectively, 99.3 and 98.7%) [5]; moreover, compared to occlusion, stenosis is an uncommon mode of early graft failure [5], [9]. MDCT examination is a relatively painless procedure when compared to invasive coronary angiography, and very good patient acceptability of coronary MDCT angiography is documented in the literature [10], [11]. It is less expensive, is rapid, utilizes fewer resources than coronary angiography and is performed in an outpatient setting. Finally, according to the American College of Cardiology, the American College of Radiology and the North American Society for Cardiovascular Imaging, CABG patency assessment with CT is an appropriate approach in symptomatic patients at risk for graft stenosis/occlusion [12].

Nevertheless, previous studies investigating the accuracy of 16- and 64-slice MDCT for graft patency showed a number of scanned grafts that were not fully assessable [5]. High and irregular heart rates are among the important factors causing degraded image quality in MDCT imaging of native coronary artery as well as of CABG.

Scanners with larger z-axis coverage and faster rotation have recently been introduced, and they offer a strong potential for improved image quality. Only few studies have reported CABG imaging with systems newer than 64-slice MDCT [13]–[16]. The main objectives of the present study are to assess the qualitative and quantitative image quality in CABG evaluation using 256-slice scanner with prospective ECG-gating, as well as the effect of heart rate, heart rate variability and z-axis location on graft image quality.

## Materials and Methods

### Study population

Our center is involved in two major studies using MDCT for the assessment of CABG techniques. 1- The AMI-PONT trial (ClinicalTrials.gov: NCT01585285) is a prospective single center trial involving consecutive patients randomized to a new composite surgical CABG design for the anterolateral coronary territory, an internal mammary artery (IMA) with saphenous vein bridge (SVB) graft (IMA-SVB), versus the conventional CABG design. As a pilot study, between June 2010 and February 2011, a subgroup of 20 patients with LIMA-SVB underwent MDCT. Patients with the longest postoperative follow-up were contacted first. All these 20 patients are included in the present study. Data on graft patency was previously published [17], but data on graft segment image quality assessment and association with heart rate and z-axis location in these patients have never before been reported. 2- The PATENCY-CORONARY trial (ClinicalTrials.gov: NCT01414049) is a multicenter prospective trial of consecutive patients undergoing CABG surgery with (on-pump) versus without (off-pump) cardiopulmonary bypass. The PATENCY-CORONARY trial is ongoing and will determine whether off-pump CABG compared to conventional on-pump CABG surgery is associated with lower CABG patency after mid-term postoperative follow-up, as assessed with MDCT. The first 58 PATENCY-CORONARY patients enrolled in our center between March 2011 and December 2011 are included in the present study.

Approval has been obtained by the Institutional Review Board of the University of Montreal Medical Center (*comité d′éthique de la recherche du CHUM*). Written informed consent was provided by all subjects before enrollment.

The total study group consists of 78 patients (71 men, 7 women; mean age of 68.6 ± 7.5 years) with CABG. They underwent MDCT graft evaluation (mean postoperative follow-up 23.5 ± 16.4 months; range 2 – 56 months) in the University of Montreal Medical Center. Patient characteristics and scan-related parameters are shown in Table 1.

**Table 1:** Patient characteristics and scan-related parameters.

**Patient characteristics (n = 78 MDCT patients with CABG)**		
Men/women		71/7
Age (years) (mean ± SD)		68.6 ± 7.5
Mean number of grafts (range)		3.2 (2–6)
Postoperative graft follow-up (months) (mean ± SD)		23.5 ± 16.4
BMI (kg/m^2^) (mean ± SD)		27.1 ± 3.9
Smoker (n, %)		15 (19)
Hypertension (n, %)		55 (71)
Hypercholesterolemia (n, %)		64 (82)
Diabetes (n, %)		20 (26)
Long-term betablocker therapy (n *) (%)		66 (84.6)
NYHA	class I n (%)	70 (89.7%)
	class II n (%)	8 (10.3%)
CCS	class I n (%)	76 (97.4%)
	class II n (%)	2 (2.6%)
Mean LVEF (mean ± SD)		56 ± 10%
**Scan-related parameters**		
Mean heart rate during scan (bpm) (mean ± SD)		59.7 ± 9.8
Heart rate variability ** during scan (bpm) (mean ± SD)		7.2 ± 1.6
Prescan betablocker administration, n (%)		37 (47.4)
Prescan nitroglycerin administration, n (%)		69 (88.5)
Contrast agent (ml) (mean ± SD)		106 ±11
Scan coverage (mm) (mean ± SD)		251.9 ± 28.7
DLP *** (mGy-cm) (mean ± SD)		705.4 ± 103.1
Effective dose (mSv) (mean ± SD)		9.9 ±1.4

Standard deviation (SD); body mass index (BMI); beats per minutes (bpm); New York Heart Association functional classification (NYHA); NYHA Class I: No limitation of physical activity; NYHA Class II: Slight limitation of physical activity; Canadian Cardiovascular Society Angina Grading Scale (CCS); CCS Class I: Ordinary physical activity does not cause angina; CCS Class II: Slight limitation of ordinary activity; Left ventricular ejection fraction (LVEF).

*: n, number of patients.

**: The standard deviation of the heart rate in a given patient is the parameter used for heart rate variability measurement.

***: DLP, dose-length product of MDCT angiography.

Exclusion criteria for MDCT investigation were severe renal impairment (glomerular filtration rate (eGFR) <35 mL/min/1.73 m^2^), atrial fibrillation, history of severe hypersensitivity to iodinated contrast agents, severe cardiorespiratory limitation (New York Heart Association (NYHA) functional Class IV or Canadian Cardiovascular Society (CCS) Angina Grading Scale Class IV), and inability to give informed consent. Patients with eGFR 35–50 mL/min/1.73 m^2^ were treated with hydration and N-acetylcysteine (Mucomyst®) and those with only mild hypersensitivity received corticosteroids before contrast administration. Cardiac symptoms were neither among inclusion nor exclusion criteria, except for symptoms of NYHA functional class IV or CCS angina class IV (Table 1).

### CT Imaging

#### Patient preparation

In patients with heart rate > 60 beats per minute (bpm), 50–75 mg of metoprolol (adapted to the patient body weight) was given orally 45–60 minutes prior to MDCT. Contraindications to beta-blockers were active chronic obstructive pulmonary disease, severe aortic valve stenosis, or systolic blood pressure < 100 mmHg. Patients also received 0.4 mg of nitroglycerin sublingually 2 minutes prior to MDCT. Contraindications to nitroglycerin were systolic blood pressure < 100 mmHg, hypersensitivity to nitroglycerin or recent use of PDE5 inhibitor, such as sildenafil, tadalafil, and vardenafil.

#### MDCT data acquisition

A 256-slice MDCT scanner was used (Brilliance iCT, Philips Healthcare, Cleveland, OH, USA). This scanner has an array of 128 detectors and allows, with image length-dependent adaptive z-collimation, 256 slices of 0,625 mm per rotation. Matrix was 512 × 512, and field-of-view 250 mm. Scan voltage was 120 kV, and gantry speed 270 ms. Prospective ECG-gating was used for all patients, targeting a physiologic diastolic phase at 75% of R-R cycle (Step & Shoot Cardiac, Philips Healthcare, Cleveland, OH, USA). In addition, a phase tolerance of ± 5% (additional buffer) was used enabling reconstruction of neighboring phases of 70% and 80%.

Scans were performed in a cephalic to caudal direction, during shallow inspiration breath-hold, with scan range from the clavicles to the lung bases, in order to visualize the heart as well as the internal mammary arteries.

#### MDCT image reconstruction and postprocessing

A slice thickness of 0.8 to 0.9 mm and mean increment of 0.45 mm was used for reconstruction of axial images, with a standard (smooth) kernel (XCB, Philips Healthcare, Cleveland, OH, USA). At the time of the study, iterative reconstruction algorithm was not available on this scanner. Filtered back projection was used for image reconstruction. CT images were then transferred to a thin client postprocessing software (Aquarius Intuition edition version 4.4.4.23.771, TeraRecon Headquarters, Forster City, CA, USA). Postprocessing features included volume-rendered, orthogonal, curved planar, and maximum intensity projection reformations.

#### Injection parameters

The contrast agent was administered with a power injector at a steady flow rate of 5 ml/sec, using iodixanol (320 mg I/mL, Visipaque 320, GE Healthcare Canada Inc., Mississauga, Ontario, Canada).

Prior to its administration, the contrast agent was pre-warmed to body temperature (37.0 °), and injected through a large angiocatheter (ideally via an 18-gauge catheter, if not via a 20-gauge catheter) securely placed in a substantial peripheral vein in or around the antecubital fossa of the right arm. The left arm was used if the right arm was not accessible. The following protocol was used: 80 ml of a solution made of 100% of the contrast agent at a rate of 5 ml/sec, followed by 55 ml of a solution made of 40% contrast agent and 60% of saline at a rate of 5 ml/sec, finally followed by 40 ml of 100% saline at a rate of 5 ml/sec. Bolus tracking was performed with a region of interest (ROI) placed in the descending aorta. Image acquisition started with a minimal delay after reaching a predefined attenuation threshold of 200 Hounsfield units (HU) in the descending aorta.

#### Radiation dose

Radiation dose was estimated based on the dose-length product (DLP) as indicated on the dose report of the CT scanner. The effective radiation dose of MDCT angiography was estimated by the product of the DLP and a conversion coefficient for the chest (k = 0.014 mSv*mGy−1*cm−1).

### Image analysis

The observers (see below) had access to the surgical coronary artery graft report, as they would in a normal clinical setting. For image analysis, the observers could use any of the image data sets (70%, 75% and 80% of the R-R interval) in all patients. Observers were blinded to the heart rate and heart rate variability data at the time of the reading.

#### Qualitative image quality assessment

For graft qualitative image quality evaluation, internal mammary artery (IMA) and aortocoronary saphenous vein (SV) grafts were divided in separate segments, according to Willmann al. [18]. The proximal segment included the proximal 1-cm length of the graft body, for IMA as well as for aortocoronary SV grafts, and the proximal anastomosis for aortocoronary SV grafts (IMA grafts were usually in situ IMA grafts). The distal segment included the distal 1-cm of the graft body, as well as the distal graft anastomosis. The middle segment included the graft body, excluding the proximal 1-cm length and distal 1-cm length of the graft.

Qualitative image quality analysis was performed separately by two independent observers (BG and PDM; 3- and 6-year experience in cardiovascular MDCT, respectively). For all patients, graft segments were assessed for image quality by both observers, and rated on a 5-point grading scale: 1, nondiagnostic (diagnostic information not obtainable); 2, poor (severe artifacts with blurring of the segment lumen); 3, moderate (moderate artifacts with moderate blurring of the segment lumen); 4, good (minor artifacts with slight blurring of the segment lumen); and 5, excellent (no artifacts with clear delineation of the entire segment lumen). Image quality degradation caused by stepladder artifacts, surgical clip artifacts, respiratory artifacts, insufficient opacification or small distal anastomosis was recorded. Conflicting results were resolved by consensus at a subsequent reading session.

Graft stenoses or occlusions were reported to the treating physician, as soon as detected, as well as any significant extra-cardiac finding.

#### Quantitative image quality assessment

One observer (BG) made quantitative measurements in the body of IMA and aortocoronary SV grafts, as well in the origin of the IMA grafts. ROIs were manually drawn centrally in the lumen of the bypass grafts, on the axial images. ROI diameter was drawn as large as possible, provided that the ROI remained intraluminal. In IMA grafts, a ROI was placed in the lumen of the body of the IMA at the level of the bifurcation of the pulmonary arteries, and another ROI in the lumen of the origin of the IMA. For aortocoronary SV graft quantitative assessment, one aortocoronary SV graft per patient was assessed, usually an aortocoronary SV graft to the right coronary artery (RCA) territory, with a ROI in the lumen of the body of the graft at the level of the bifurcation of the pulmonary arteries. Vessel wall as well as atherosclerotic plaques and calcifications were excluded of all ROI's.

Attenuation, image noise, and signal-to-noise ratio (SNR) were assessed in IMA and aortocoronary SV grafts, using ROI placement as described above. Image noise was defined as the ROI attenuation standard deviation (HU), and SNR was calculated as the mean attenuation value of a particular ROI divided by image noise of this same ROI.

CNR of the left ventricle (LV-CNR) was assessed by using manually drawn ROI's in the contrast-enhanced left ventricular chamber and the free wall of the left ventricular myocardium. LV-CNR was defined as the difference between of mean attenuation values of the contrast-enhanced left ventricular chamber and the left ventricular wall divided by image noise.

### Heart rate and heart rate variability

The heart rate and the heart rate variability were evaluated using semiautomatic software on the workstation (Extended Brilliance Workspace 3.5, Philips Healthcare, Cleveland, OH, USA). The workstation provides a mean value for heart rate during ECG-gated CT acquisition, as well as the standard deviation of the heart rate. The standard deviation of the heart rate in a given patient was the parameter used for heart rate variability measurement.

### Statistical analysis

Continuous data were expressed as mean value ± standard deviation (SD) or otherwise stated. Categorical data were expressed as proportions.

For analyses of the association between heart rate, heart rate variability, BMI, age, use of prescan nitroglycerine, LVEF and the graft qualitative image quality scores, a logistic mixed model was used with random effects on patients to account for repeated measures on the same patient and nested effects on graft types. For analyses of quality scores involving the association between graft type and graft segment location along the z-axis, cumulative link mixed models were used, with arterial versus venous graft status represented by a binary indicator with arterial graft as the reference category, and with location represented by two binary indicators (body and distal locations) with proximal location as the reference category.

For within-patient comparison of normally distributed continuous variables, the Student's paired t-test was used. For non normally distributed variables, a Friedman test was used when applicable, followed by Wilcoxon signed-rank tests, with Bonferroni adjustment for multiple comparisons. Pearson or Spearman coefficients were used for correlation between BMI and other continuous variables, for data with normal and non normal distribution, respectively. Mann-Whitney U test was used for comparison of noise between patients with low and high BMI.

Agreement between initial evaluations for graft patency assessment by the two observers was assessed by the weighted Cohen's kappa statistic for ordinal variables [19]. A kappa value of less than 0.20 implied poor agreement; 0.21–0.40, fair agreement; 0.41–0.60, good agreement; 0.61–0.80, very good agreement; and 0.81–1.00, excellent agreement [20].

A two-tailed p value < 0.05 was considered statistically significant. Statistical analyses were performed using SPSS (SPSS versions 17 and 21, Inc, Chicago, Illinois) and R [21], [22].

## Results

### Patient population

A total of 78 patients with a mean postoperative follow-up of 23.5 ± 16.4 months (range 2–56 months) were included in the study (Table 1). MDCT CABG examinations were successfully completed in all patients. No graft was excluded from the analysis.

#### Graft characteristics

**Figure 1:**
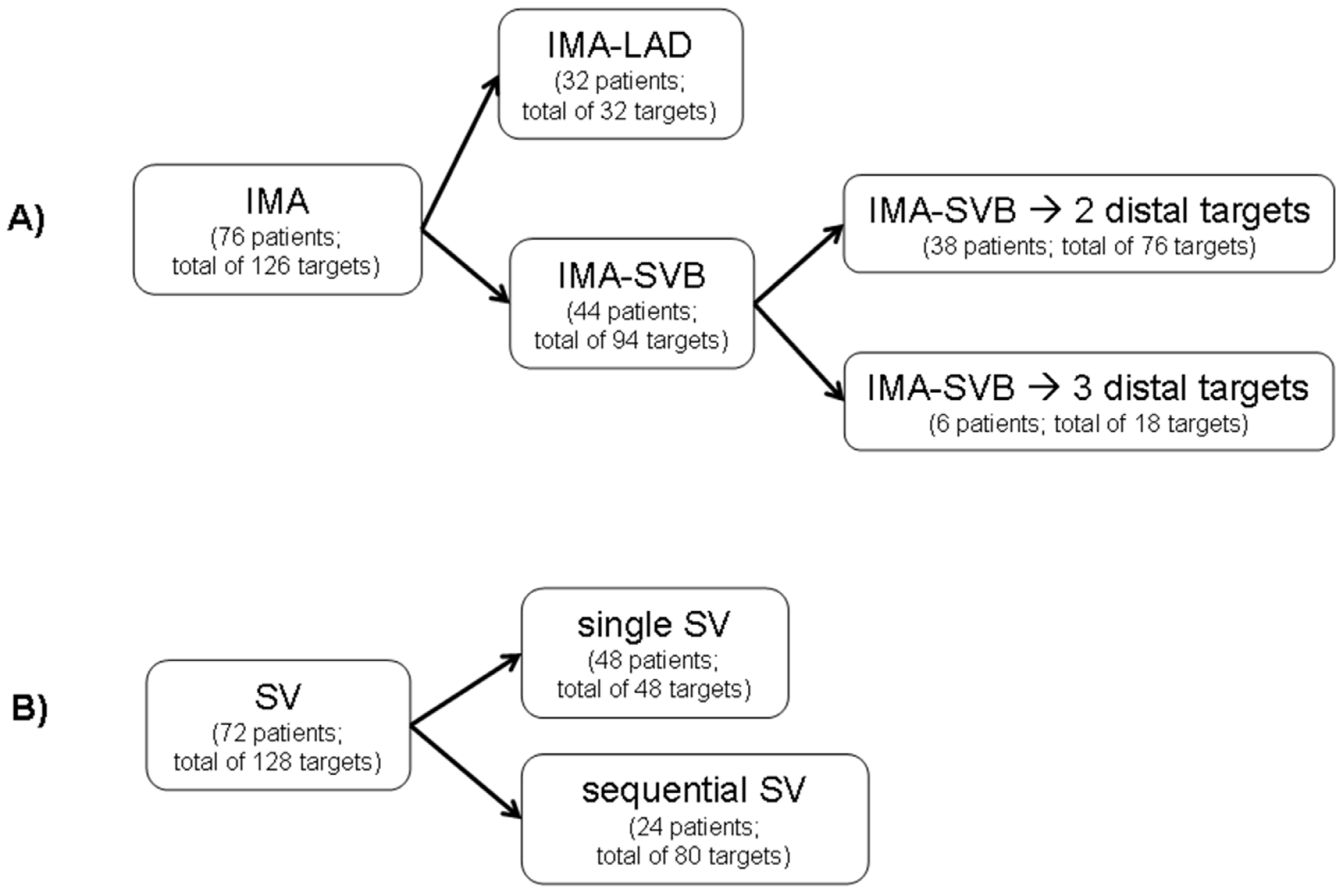
Coronary artery bypass grafts in the 78 patients of the study. Distribution of A) internal mammary artery (IMA) grafts and B) saphenous vein (SV) grafts in 78 patients with a total of 254 coronary artery bypass grafts (CABG) scanned with 256-slice MDCT. IMA-LAD indicates simple IMA grafts to the left anterior descending artery, and IMA-SVB, composite IMA with saphenous vein bridge grafts. An IMA-SVB graft redistributes the flow of the IMA to two or more coronary arteries (distal targets) of the anterolateral territory, usually to the left anterior descending artery as well as to one or more diagonal arteries, or to the ramus intermedius artery. A single SV graft has a proximal anastomosis on the ascending aorta, and a distal terminolateral anastomosis on one distal target. A sequential SV graft has a proximal anastomosis on the ascending aorta, and sequential distal anastomoses on two (or more) distal targets: one (or more) laterolateral anastomosis, and a most distal one which is terminolateral. Most patients had both an IMA graft (used as a simple or composite graft) and one (or more) SV graft(s).

The 78 patients had a mean of 3.2 bypass grafts (range 2–6 bypass grafts) (Table 1). A total of 254 grafts were available for evaluation (126 arterial grafts and 128 venous grafts). Figure 1 describes the different graft types of the patients. Most patients had both an IMA graft and one (or more) SV graft(s).

Among the 254 grafts, there were 107 grafts to the LAD territory (42.1%), 93 to the circumflex territory (36.6%) and 54 to the RCA territory (21.3%). Overall, as based on the CT findings, the lumen of 224 (88.2%) grafts among the 254 grafts was either normal or with a stenosis < 50% in diameter. There were 14 (5.5%) grafts with a stenosis ≥ 50% in diameter, and 16 grafts (6.3%) were occluded.

#### CT examination characteristics

Average mean heart rate during the scan was 59.7 ± 9.8 beats per minutes (range 38–98 beats per minutes; 25^th^ to 75^th^ percentiles, 53–65 bpm; 10^th^ to 90^th^ percentiles, 49.9–71.2 bpm). See also Table 1 for other scan-related parameters.

### Qualitative image quality

A total of 762 (100%) bypass graft segments were evaluated by both observers for subjective image quality assessment. Interobserver agreement was excellent, with kappa values of 0.90, 0.90 and 0.94 for simple IMA, aortocoronary SV and IMA-SVB grafts, respectively (p < 0.05).

**Figure 2:**
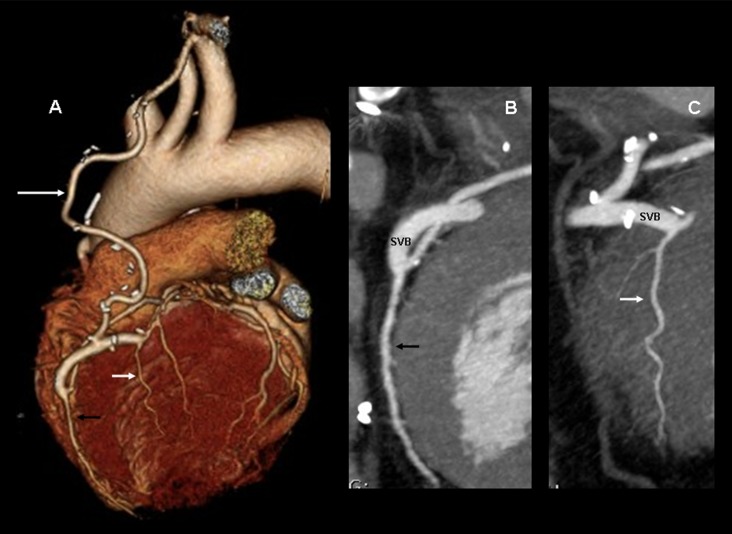
Composite coronary artery bypass graft. 256-slice MDCT with prospective ECG-gating, volume rendering (A) and curve reformat (B, C) of a 56-yo man with a composite internal mammary artery with saphenous vein bridge graft (IMA-SVB). The IMA-SVB graft redistributes the flow of the IMA (long white arrow) to the left anterior descending artery (LAD) (black arrow) and to the first diagonal artery (D1) (short white arrow). Distal graft anastomoses on LAD and D1 are well seen. The scores for image quality in all graft segments were 5 (excellent), for both 2 independent observers. SVB: saphenous vein bridge.

**Figure 3:**
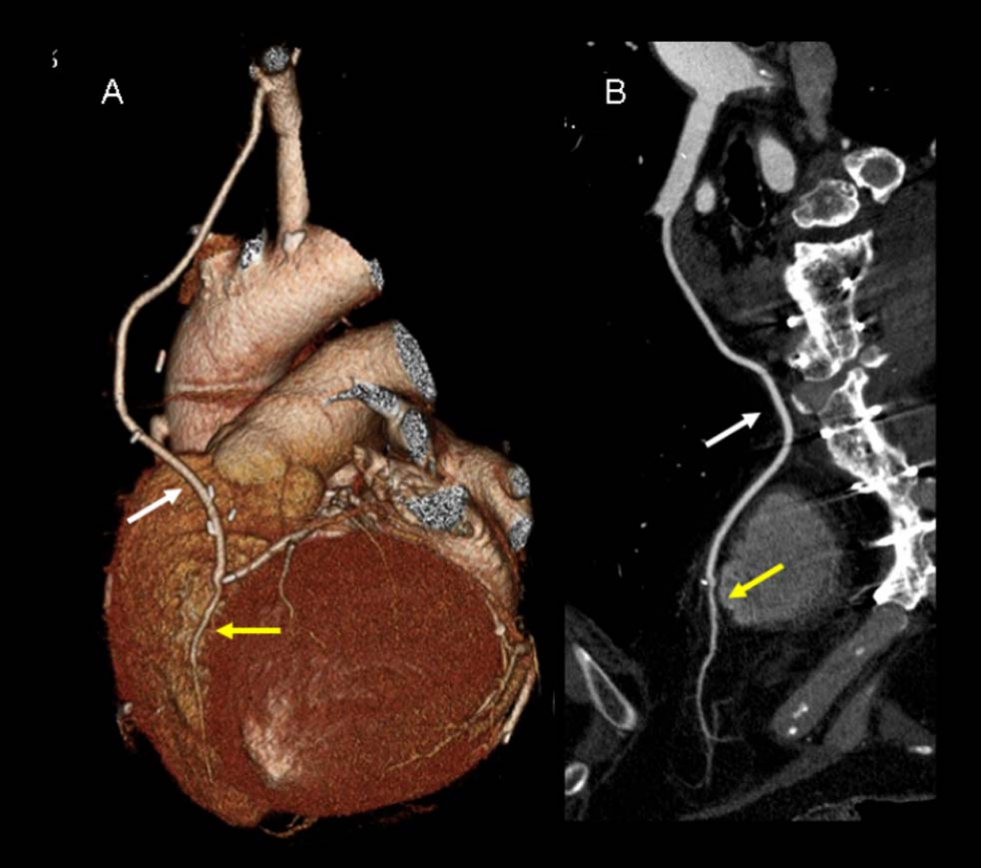
Coronary artery bypass graft – Non significant metallic artifacts. 256-slice MDCT with prospective ECG-gating, volume rendering (A) and curve reformat (B), of a 77-yo man with a patent left internal mammary artery graft (IMA) (white arrows) on the left anterior descending artery (yellow arrows) (postoperative follow-up of 14 months). The scores for image quality in proximal, middle and distal graft segments were 5 (excellent), 5 and 5, respectively, for both 2 independent observers. Neither the artifacts from the surgical clips near the IMA (A) or from the sternal metallic wires (B) were reported as significant by any of the observers. Mean heart rate was 38 bpm. Heart variability (standard deviation of heart rate) was 1.0 bpm.

Table 2 shows the distribution of the qualitative scores for the 762 graft segments, after consensus. Among the 762 segments, 69.9% of segments were associated with score 5 (excellent), 22.6% with score 4 (good), 4.1% with score 3 (moderate), 2.4% with score 2 (poor), and 1.0% with score 1 (nondiagnostic) (Figure 2). Degraded image quality in the 26 segments (11 patients) with scores 1 and 2 was related to stepladder artifacts (n = 6), poor opacification (n = 5), small distal anastomosis and coronary artery bed (n = 5), clips artifacts (n = 5), motion artifacts (blurring) (n = 4) and graft segment tortuosity (n = 1) (Figure 3). Average mean heart rate and average heart rate variability for these 11 patients with segment scores 1 or 2 were 66 and 8.2 bpm, respectively (range 50–81 and 0.6–33.2, respectively).

**Table 2:** Qualitative image quality graft segment score distribution in 254 coronary artery bypass grafts from 78 patients.

	Graft segments * (n, %)	Normal ** graft segments (n, %)	Stenotic *** graft segments (n, %)	Occluded graft segments (n, %)
Score 5 (excellent)	533 (69.9)	495 (73.7)	20 (47.6)	18 (37.5)
Score 4 (good)	172 (22.6)	145 (21.6)	7 (16.7)	20 (41.7)
Score 3 (moderate)	31 (4.1)	24 (3.6)	4 (9.5)	3 (6.3)
Score 2 (poor)	18 (2.4)	6 (0.9)	11 (26.2)	1 (2.1)
Score 1 (nondiagnostic)	8 (1.0)	2 (0.3)	0 (0)	6 (12.5)
Marginal sums (%)	762 (100)	672 (88.2)	42 (5.5)	48 (6.3)

Distribution of consensual qualitative image quality graft segment scores (scores 1 to 5) in 254 coronary artery bypass grafts from 78 patients, as evaluated with 270-msec gantry rotation 256-slice MDCT and prospective ECG-gating. Score distribution is shown for the overall 762 graft segments, then separately for the 672 normal graft segments, the 42 stenotic graft segments, and the 48 occluded graft segments. Score definition: 1, nondiagnostic (diagnostic information not obtainable); 2, poor (severe artifacts with blurring of the segment lumen); 3, moderate (moderate artifacts with moderate blurring of the segment lumen); 4, good (minor artifacts with slight blurring of the segment lumen); and 5, excellent (no artifacts with clear delineation of the entire segment lumen).

n: number;

*: overall graft segments (either normal, ≥ 50% stenotic, or occluded).

**: graft segments with none or < 50% stenosis.

***: graft segments with stenosis ≥ 50%.

Table 2 also shows the score distribution in graft segments with either normal, stenotic or occluded lumen. The score distribution of normal graft segments showed a strong skewness toward high quality scores (scores 4 and 5), in contrast to the more homogeneous distribution of stenotic and occluded graft segments scores.

#### Image quality: effect of heart rate, heart rate variability, BMI, age, nitroglycerin and LVEF

**Figure 4:**
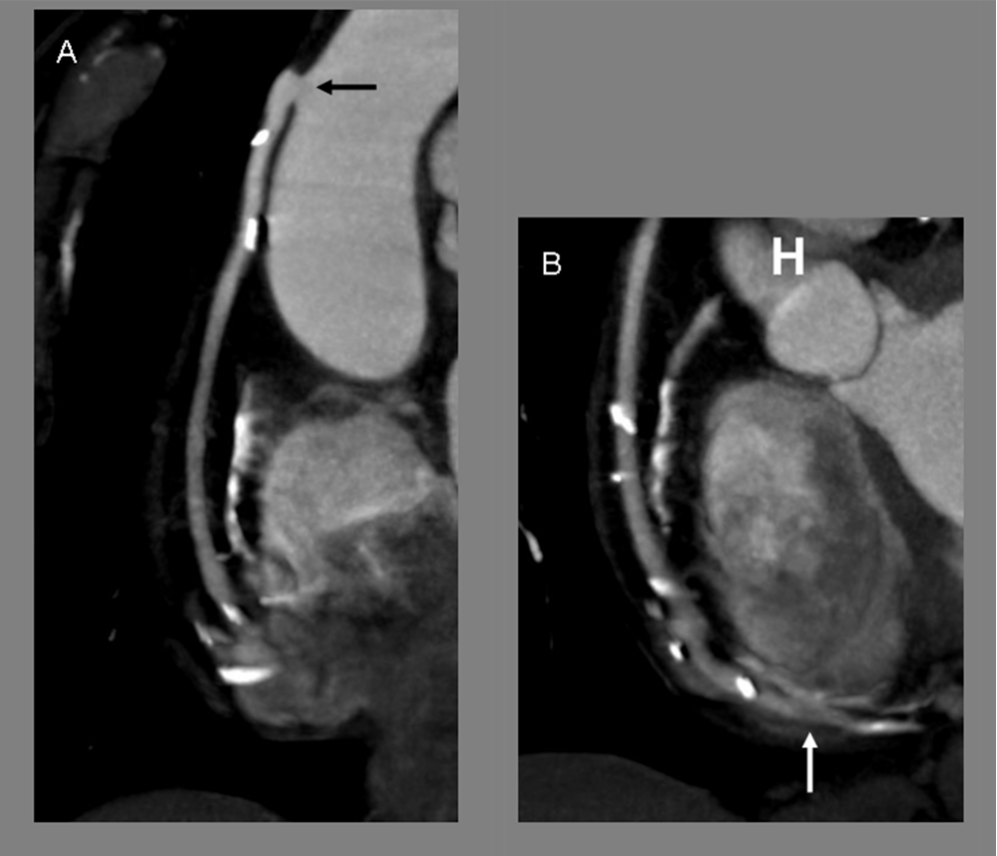
Coronary artery bypass graft – Significant cardiac motion artifacts. 256-slice MDCT with prospective ECG-gating, curve reformat (A, B), of a 79-yo woman with a sequential aortocoronary saphenous vein graft to the distal right coronary artery, and then to the first marginal artery of the circumflex artery. A, B: the first graft sequence is shown, from the lateroterminal proximal anastomosis (black arrow) on the ascending aorta, to the laterolateral distal anastomosis (white arrow) on distal right coronary artery. The scores for image quality in proximal, middle and distal graft segments were 5 (excellent), 3 (moderate) and 3, respectively, for both 2 independent observers. Mean heart rate was 65 bpm. Heart variability (standard deviation of heart rate) was 4.9 bpm.

For analyses of the association between mean heart rate, heart rate variability, BMI, age, use of prescan nitroglycerin and LVEF, and quality scores, a mixed logistic regression model was used, to account for repeated observations on each patient. Scores 1 to 3 were considered as low scores, and were compared to higher scores (scores 4 and 5). Low quality scores were significantly associated with heart rate variability ≥ 1 bpm, with an odds ratio of 4.31 (95% confidence interval (CI) 1.10–16.84; p  =  0.036) (Figure 4). Association between low quality scores and BMI was near significance level (p  =  0.053), with an odds ratio of 1.15 (95% CI 1.00–1.32). There was no significant association between image quality scores and heart rate level, and neither with other predictor variables (Table 3).

**Table 3:** Association between heart rate, heart rate variability, BMI, age, use of prescan nitroglycerin and LVEF, and low image quality graft segment consensual scores (score 1 to 3).

	OR	CI 95%	p value
Mean heart rate	0.98	0.92–1.04	0.439
Heart rate variability ≥ 1 bpm	4.31	1.10–16.84	0.036
BMI	1.15	1.00–1.32	0.053
Age	1.03	0.95–1.12	0.455
Prescan nitroglycerin	0.60	0.10–3.72	0.580
LVEF ≥ 60%	0.87	0.22–3.46	0.847

OR: odds ratio; CI 95%: confidence intervals 95%; BMI: body-mass index; LVEF: left ventricular ejection fraction.

Results of logistic regression analysis.

#### Image quality: effect of z-axis location of the graft segment

The association between the graft segment location along the z-axis (proximal, middle or distal) and qualitative image quality was assessed on 177 grafts (531 graft segments). Grafts with transverse orientation (distal sequences of sequential aortocoronary SV grafts, and SVB segments of the IMA-SVB composite grafts) were not included in this analysis.

**Figure 5:**
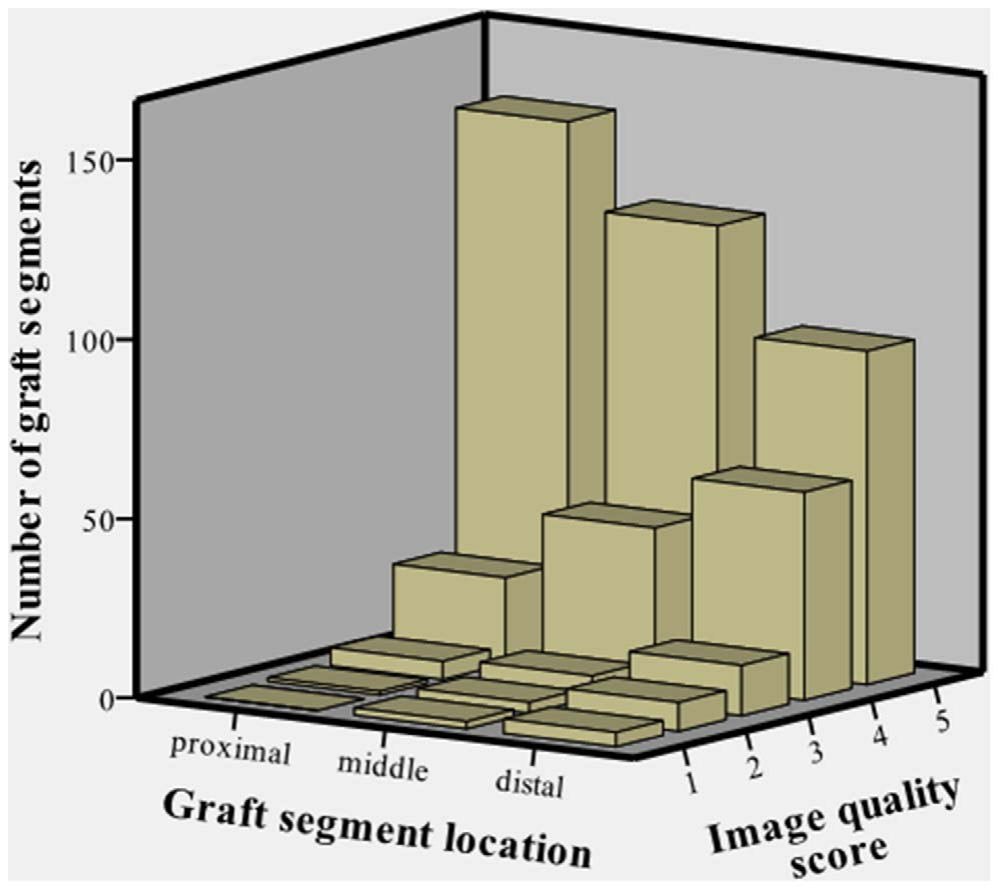
Distribution of image quality score according to coronary artery graft segment location. Bar chart shows the analysis of the image quality score distribution for proximal, middle and distal segments of 177 grafts with cephalic to caudal orientation (531 graft segments). Distal graft segments were associated with significantly lower image quality scores than proximal segments.

Table 4 provides the image quality score distribution by graft segment location (proximal, middle and distal) (Figure 5). Quality scores were in the diagnostic range (scores 3, 4 and 5) in 99.4% of proximal graft segments, as well as in 97.2% and 93.2% of middle and distal graft segments, respectively.

**Table 4:** Image quality score distribution by graft segment location.

	Graft segment location		
	Proximal	Middle	Distal
**Score (n, %)**			
1	0 (0)	2 (1.1)	4 (2.3)
2	1 (0.6)	3 (1.7)	8 (4.5)
3	5 (2.8)	6 (3.4)	14 (7.9)
4	24 (13.6)	43 (24.3)	58 (32.8)
5	147 (83.0)	123 (69.5)	93 (52.5)

The association between the graft segment location along the z-axis (proximal, middle or distal) and qualitative image quality was assessed on 177 grafts (531 graft segments). Grafts with transverse orientation (distal sequences of sequential aortocoronary SV grafts, and SVB segments of the IMA-SVB composite grafts) were not included in this analysis.

n: number of segments.

**Figure 6:**
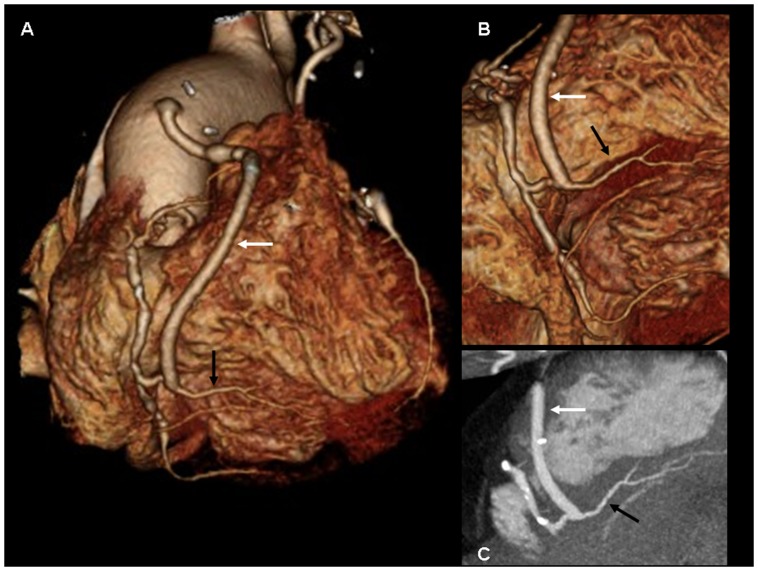
Coronary artery bypass graft – Terminolateral distal anastomosis. 256-slice MDCT with prospective ECG-gating, volume rendering (A,B) and maximum intensity projection (C), of a 56-yo man with a aortocoronary saphenous vein graft (white arrow) to the posterior descending artery (PDA) (black arrow). An excellent opacification of a normal distal graft anastomosis on PDA can be seen.

In venous grafts, mean score (4.82 ± 0.52) in proximal segments was significantly higher compared to middle segments (4.49 ± 0.77) (p < 0.0001), and mean score in middle segments was significantly higher compared to distal segments (4.24 ± 0.99) (p = 0.02) (Figure 6). In IMA grafts, proximal (4.75 ± 0.49) and middle (4.74 ± 0.68) segment mean scores were similar (p = 0.76), and both were higher than distal segment mean score (4.36 ± 0.92) (both p <0.0005) (Table 5).

**Table 5:** Mean image quality score (SD) by graft segment location.

	Grafts type			
	Venous		IMA	
**Segment location**				
Proximal	4.82*	(0.52)	4.75***	(0.49)
Middle	4.49**	(0.77)	4.74***	(0.68)
Distal	4.24	(0.99)	4.36	(0.92)

SD, Standard deviation

*: In venous grafts, mean score in proximal segments was higher compared to middle segments (p < 0.0001);

**: In venous grafts, mean score in middle segments was higher compared to distal segments (p = 0.02);

***: In IMA grafts, both proximal and middle segment mean scores were higher than distal segment mean score (p <0.0005).

### Quantitative image quality

Quantitative measurements were made in middle segments of aortocoronary SV and IMA grafts, and are described in Table 6. Mean values of attenuation, noise and SNR were significantly better in aortocoronary SV grafts in comparison to IMA grafts (p < 0.002, p < 0.001, and p < 0.001, respectively).

**Table 6:** Mean quantitative image quality graft parameters – Comparison between aortocoronary saphenous vein and internal mammary grafts (middle segments).

	Mean ± SD		
	Aortocoronary SV grafts	IMA grafts	P value
Attenuation (HU)	415.5 ± 66.4	359.2 ± 89.1	< 0.002
Aortocoronary SV grafts noise (HU)	36.1 ± 29.2	44.3 ± 21.5	< 0.001
Aortocoronary SV grafts SNR	15.8 ± 7.9	10.3 ± 6.4	< 0.001

SD: standard deviation; SV: saphenous vein; IMA: internal mammary artery; HU: Hounsfield units; SNR: signal-to-noise ratio.

The origin of the IMA is at the same level as the shoulders, where more streak artifacts occur. For this reason, quantitative measurements were also made at the origin of the IMA grafts, for comparison with the IMA graft middle segment. The results are described in Table 7. Mean value of noise was significantly higher at the origin of the IMA grafts compared to their middle segment (p  =  0.001). No statistically significant difference was shown for mean values of attenuation and SNR.

**Table 7:** Mean quantitative image quality graft parameters – Comparison between the origin and the middle segment of internal mammary grafts.

	Mean ± SD		
	IMA graft origins	IMA graft middle segments	P value*
Attenuation (HU)	370.9 ± 79.1	359.2 ± 89.1	0.30
Noise (HU)	58.4 ± 30.0	44.3 ± 21.5	0.001
SNR	8.6 ± 5.2	10.3 ± 6.4	> 0.05

SD: standard deviation; IMA: internal mammary artery; HU: Hounsfield units; SNR: signal-to-noise ratio.

*: with Bonferroni adjustment.

Mean BMI was 27.1 ± 3.9 kg/m^2^. Twenty-five patients (32.1%) had a BMI ≤ 25 kg/m^2^, and 53 (67.9%) a BMI > 25 kg/m^2^. BMI correlated negatively with aortocoronary SV lumen attenuation (r  =  −0.44; p < 0.001), with LV chamber attenuation (r  =  −0.29; p < 0.01), and with LV CNR (r  =  −0.55; p < 0.001). No significant correlation was found between BMI and IMA lumen and LV wall attenuation, neither with heart rate or heart rate variability. Noise at the IMA origin was not significantly higher with BMI > 25 kg/m^2^, in comparison to BMI ≤ 25 kg/m^2^ (p  =  0.15).

## Discussion

In this study, a total of 78 patients with 254 CABG (762 graft segments) were recruited to undergo CABG assessment with 270-msec rotation prospectively ECG-gated 256-slice MDCT. Image quality was judged of diagnostic quality in 96.6% of segments, and as poor or non-diagnostic in 3.4% of them. Interobserver agreement was excellent. Graft image quality was not influenced by the level of heart rate. Our results however showed that image quality scores were significantly better in patients with low heart rate variability (odds ratio 4.31; p  =  0.036). A high BMI also induced a lower image quality, although to a milder degree (odds ratio 1.15; p  =  0.053). Finally, distal graft segments were associated with significantly lower image quality scores than proximal segments.

With 16- and 64-slice MDCT, CABG are not fully assessable in up to 22% of cases [5]. CABG imaging with scanners newer than 64-slice MDCT has been assessed in only few studies, with improved results. With 64-slice and 128-slice dual-source MDCT, rate of inadequate image quality was as low as 0.6 to 2% [13] [14]. With the use of 256-slice MDCT, Lee et al. [15] studied 34 CABG patients with retrospective EGC-gating, and 30 patients with prospective ECG-gating. All graft segments were considered assessable, however after exclusion of occluded grafts from the image quality assessment. With 320-slice MDCT, de Graaf et al. [16] reported that 2.2% of grafts were non assessable, however after excluding 2 out of 40 patients from the analysis, because of non-diagnostic global image quality. In our study, using 256-slice MDCT, all 762 graft segments in 78 patients were evaluated and included in the analysis. Image quality scores were excellent in 69.9%, good in 22.6%, and moderate in 4.1%, of these 762 graft segments. Image quality was poor or non-diagnostic in only 3.4% of the graft segments. Reasons for degraded image quality in our study were stepladder artifacts, poor opacification, small graft segment caliber, surgical clips, motion artifacts and vessel tortuosity.

In native coronary artery imaging studies with 64-MDCT, dual-source MDCT, and 256-slice MDCT, image quality was worse in patients with high heart rate [23], [24] [25] [26], as well as in 64-slice MDCT imaging studies assessing CABG patients [27], [28]. Goetti at al. [14] also reported that high heart rate was associated with lower graft image quality using 128-slice dual-source MDCT in CABG patients, although this was not statistically significant. In the study of Lee et al. [15] with 256-slice MDCT, prospective ECG-gating on 30 patients with CABG was significantly associated with image quality degradation with higher heart rate. In contrast, in our study of CABG imaging with 256-slice MDCT and prospective ECG-gating, high heart rate was not associated with degraded graft image quality.

High heart rate variability has been shown to cause significant native coronary artery image quality degradation with 64-slice MDCT [23], [29] and dual-source CT [25]. CABG imaging using 64-slice MDCT also demonstrated that higher heart rate variability results in lower graft image quality [28]. In contrast, Goetti et al. [14] using 128-slice dual-source MDCT for CABG imaging unexpectedly found that high heart rate variability was associated with superior graft image quality, although this was not statistically significant. Our study with 270-msec rotation 256-slice MDCT shows that high heart rate variability results in significant and important graft image quality deterioration.

Decreased MDCT assessability of distal graft segments has been reported with 16-slice [30] and 64-slice MDCT [31], as well as with 128-slice dual-source MDCT [14]. In the subgroup of 30 CABG patients who underwent 256-slice MDCT with prospective ECG-gating, Lee et al. [15] also showed that the image quality was generally degraded as the segment approached to the distal anastomosis, although this was not stated as significant. The decreased evaluability of distal graft segments may be related to local cardiac motion, as well as to a slightly reduced contrast enhancement in the distal graft segments, due to the longer z-axis coverage required in MDCT graft imaging and the longer scan time associated with prospective ECG-gating. Our study, using 256-slice MDCT with prospective ECG-gating, also showed statistically significantly lower qualitative scores in the distal segments, which are in the vicinity of the heart. This finding is important, since a consistent part of stenoses are located near the distal graft anastomosis. Beta-blockers should probably be still considered for CABG imaging, to obtain better control on heart rate variability and image quality, especially for distal graft segments.

Since CABG imaging requires to include the IMA from its origin, MDCT acquisition must start near the supraclavicular region, where photon starvation (or streak) artifacts are produced from the shoulders. In our study, quantitative measurements showed significantly more noise at the origin of the IMA, in comparison to IMA body. CT image reconstruction in our study was done using the conventional filtered back projection method. Evidence suggests that the recently introduced CT image reconstruction method called iterative reconstruction can prevent streak artifacts [32]. Future studies should evaluate how this method could reduce noise during CABG MDCT imaging, especially at the level of the origin of the IMA, as well as how it could improve noise adjustment in patients with high BMI.

To our knowledge, our study is the largest study to assess 256-slice MDCT for CABG evaluation. MDCT imaging of CABG requires longer coverage than imaging of the native coronary arteries. This paper shows how well large-coverage MDCT scanner and step-and-shoot ECG-gating can perform in this setting, in terms of image quality and lumen visualization. Nevertheless, the study has some limitations. First, our MDCT results were not correlated with catheter coronary angiography, and the study does not assess the association between graft image quality and graft stenosis/occlusion detection, neither the diagnostic accuracy of MDCT for the evaluation of graft patency. Second, image quality of native coronary artery distal bed was not included in the analysis. Third, although we used prospective ECG-gating to decrease radiation dose, additional radiation dose reduction methods could have been used, such as lower kV, radiation dose optimisation according to patient BMI and iterative image reconstruction algorithms. These methods could be assessed in future studies with CABG patients.

## Conclusion

Coronary artery bypass graft imaging with 270-msec rotation 256-slice MDCT and prospective ECG-gating showed an adequate image quality in 96.6% of graft segments, and an excellent interobserver agreement. Graft image quality was not influenced by heart rate level. Image quality scores were however significantly decreased in patients with high heart rate variability, as well as in distal graft segments, which are closer to the heart.
