# Correction: 256-Slice CT Angiographic Evaluation of Coronary Artery Bypass Grafts: Effect of Heart Rate, Heart Rate Variability and Z-Axis Location on Image Quality

**DOI:** 10.1371/journal.pone.0100706

**Published:** 2014-06-18

**Authors:** The PLOS ONE Staff

The following Acknowledgements statement is missing from the published article:

The authors thank Mani Vembar, CT Clinical Science, Philips Healthcare, Cleveland, Ohio for his comments and suggestions in the preparation of this manuscript.
